# Biomarkers as predictive tools to test the *in vivo* anti-sarcoptic mange activity of propolis in naturally infested rabbits

**DOI:** 10.1042/BSR20180874

**Published:** 2018-12-07

**Authors:** Dina M. Metwally, Ebtesam M. Al-Olayan, Reem A. Alshalhoop, Shatha A. Eisa

**Affiliations:** 1 Department of Zoology, Faculty of Science, King Saud University, Riyadh, Saudi Arabia; 2 Department of Parasitology, Faculty of Veterinary Medicine, Zagazig University, Zagazig, Egypt; 3 National Health Laboratory, Riyadh, Saudi Arabia

**Keywords:** Propolis ointment, Rabbits, Sarcoptes scabiei, Specific biomarkers

## Abstract

The present study was designed to investigate the use of specific biomarkers, such as albumin, serum total protein, aspartate amino transferase (AST), globulin, alanine amino transferase (ALT), serum cortisol and alkaline phosphatase (ALP), as predictive tools for sarcoptic mange in rabbits. A total of 40 naturally infested rabbits were equally divided into four groups.Thirty infested rabbits were administered with three different treatments (propolis,ivermectin, and propolis with ivermectin) and were compared to10 infested un-treated rabbits. The impact of treatment was assessed via microscopic examination of skin scrapings, clinical signs, and blood measurements relating to the liver. The present study demonstrated that topical application of 10% propolis ointment resulted in complete recovery from clinical signs and complete absence of mites based on microscopic examination after 10–15 days of treatment. Moreover, AST, ALP, ALT, and cortisol were determined to be acceptable biomarkers to track the response of diseased rabbits to the therapeutic use of propolis.

## Introduction

Mange triggered by *Sarcoptes scabiei* var. *cuniculi* remains a common occurrence among pet rabbits. This parasite is unique in that it is able to inhabit the epidermal layer of the skin where the larvae’s feeding behavior and nymphs lead to hypersensitivity reactions, hyperkeratosis, inflammation, alopecia and seborrhea [1,2]. Infestation lowers animal productivity along with the overall quality of animal-based products. If left untreated, mange can be fatal [3]. Additionally, some biochemical and hematologic indices are also affected, including heightened albumin levels, serum total protein, AST, globulin, ALP and ALT [4], in addition to increased serum cortisol levels [5]. It is not as easy to eradicate *S. scabiei* in rabbits compared with other domestic animals [6]. Nonetheless, success had been attained using treatments that involve several acaricides such as deltamethrin, diazinon, and ivermectin [7]; the majority of these chemical acaricides produce side effects [8] including resistance to drugs [9], toxicity and other symptoms [10], and environmental pollution [10,11]. Therefore, new alternative treatments have been increasingly looked at with great interest [12–15]. Propolis (bee glue) is a resinous hive product collected from numerous plant sources that are popular in the folk medicine of different nations. Propolis containing products have been marketed for human use for different purposes [16], and researchers have been involved in the study of isolated compounds responsible for propolis’ therapeutic actions. Propolis is known to be replete with several pharmacological and biological properties, which have been extensively probed *in vivo* and *in vitro* [2,17]. More specifically, propolis has been used to treat common upper respiratory tract infections, infestations that bear resemblance to the flu, the common cold, and dermatological preparations to heal wounds [18]. Topical applications have been used for other ailments as well [19]. Recently, attention has been focused on the anti-parasitic activity of propolis [20–23]. The current investigation evaluated the *in vivo* acaricidal efficacy of 10% propolis ointment and ivermectin as acaricides against *S. scabiei* var. cuniculi and their effects on biochemical parameters of rabbits naturally infested with sarcoptic mites.

## Materials and methods

### Laboratory examination

In the present study, skin scrapings were gathered in a smooth manner using a scalpel blade that was dipped in mineral oil. Subsequently, samples were examined under a stereomicroscope to evaluate the mites’ mobility and morphology [24,25].

### Propolis preparation

Propolis samples were collected from beekeepers in different regions of Riyadh, Saudi Arabia in January 2016. Each sample was weighed and then frozen at −20°C, ground using a mortar, and eventually stored at 4°C until used [26]. The preparation of propolis extract was prepared as described in the literature [19] with a few modifications. We heated propolis extract (5 g) in a water bath using petroleum jelly (45 g) until mixing and melting occurred. The 10% propolis ointment prepared was then applied twice daily on the infested rabbits’ skin lesions.

### Experimental animals

A total of 40 New Zealand female rabbits weighing 2–2.4 kg ± 2.15 g were bought from Riyadh, Saudi Arabia. They were naturally infested and scored according to [27] the following indexes: 1: mild lesions (with a diameter 0–4 cm); 2: moderate lesions (with a diameter 4–8 cm); 3: severe lesions (extreme skin lesions, bloody injuries of the skin arising due to rubbing, and worsening general body condition); and 4: chronic lesions. Infestations were identified by examining the mites’ mobility and morphology using a microscope [28]. The animals were housed in wire-bottom cages within a room under generally accepted illumination conditions at 25°C ± 1°C checked daily for health and mortality for a period of 1 week until the start of the study, at which time the rabbits were divided into four groups of 10. Group I consisted of positive control (untreated) infested animals. Group II included infested rabbits treated using 10% propolis ointment twice a day. Group III included infested rabbits treated with 1% ivermectin sterile solution [Jaapharm Canada Inc., Jaamectin TM] at an SC dosage of 400 µg/kg (two injections at a 2-week interval) [4]. Finally, group IV consisted of infested rabbits treated with both ivermectin and propolis ointment, as previously described. Skin scrapings were collected on a weekly basis from the infested/recovered regions of each animal and were microscopically examined for mite detection during the course of the experiment.

### Biochemical analysis

To assess the toxic effects of propolis in the livers and kidneys of the rabbits, blood samples were taken from each rabbit’s ear vein on the 1^st^, 15^th^ and 28^th^ day to obtain serum that was subsequently stored at −20°C until evaluated. Biochemical parameters such as albumin, serum total protein, globulin, AST, ALP, and ALT [4] were evaluated using commercial kits with a Reflotron® Plus system; serum cortisol levels [5] were measured using commercially available coated-tube radioimmunoassay kits. The values prior to and post-treatment were statistically assessed.

### Statistical analysis

Using SPSS (Statistical Package for Social Sciences software; ver.22), the data were presented in the form of mean and standard error. All statistical comparisons between treated and control group were completed using a one-way ANOVA (analysis of variance) subsequently followed by a Dunnett post hoc test to make multiple comparisons. Significance was assigned at *P≤* 0.05, and an ROC (receiver operating characteristic) curve analysis was carried out. The area under the curve (AUC), degree of specificity, cut-off values, and sensitivity were subsequently calculated.

### Ethical approval

All experiments were carried out as per the specifications of the animal ethics committee outlined by the University of Sattam Bin Abdulaziz University (IRB number:**SAU-2016-LAB-455/PI**), including the joint efforts of the Parasitology Department, Sattam Bin Abdulaziz University, and the College of Science, King Saud University.

## Results

### Laboratory examination and macroscopic evaluation

**Figure 1:**
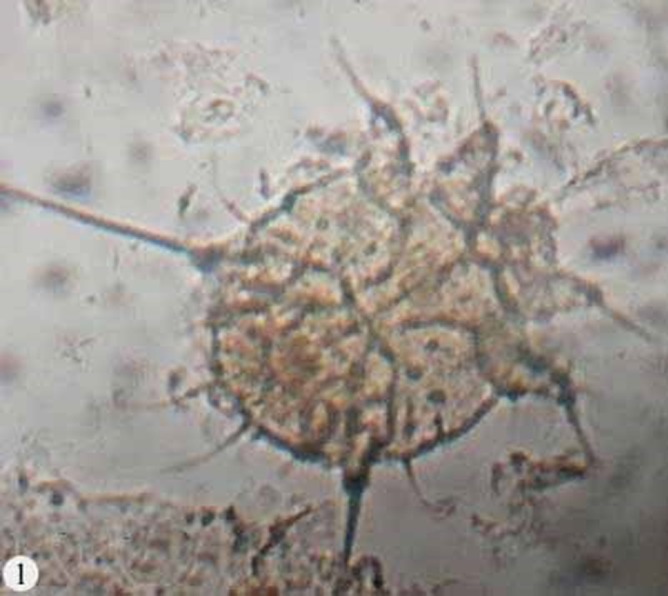
Male *Sarcoptes scabiei* (ventral view) (×400)

*S. scabiei* var. *cuniculi* were collected from the skin lesions of naturally infested rabbits (Figure 1). Since the adult stages have eight legs, they were easily distinguished from larvae and nymphs having six legs.

**Figure 2:**
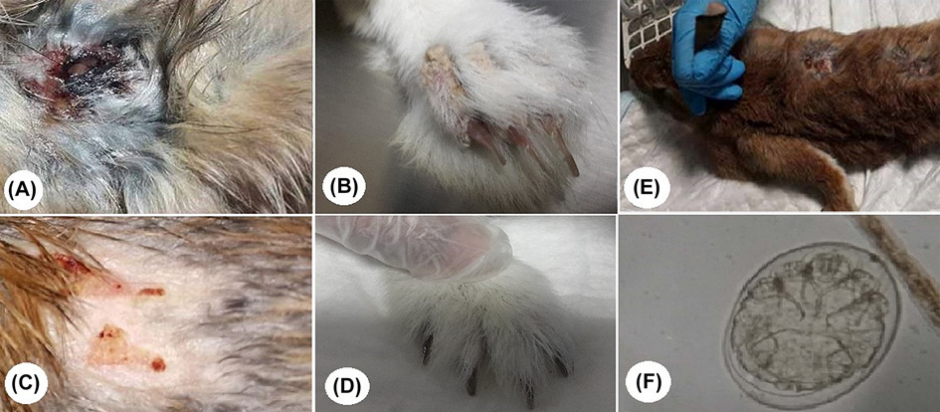
Rabbits infested with *S. scabiei* var*. cuniculi* (**A**) Body surface and (**B**) legs of rabbit infested with *S. scabiei* before treatment. (**C**) Recovery at 10 days after treatment with propolis ointment. (**D**) Recovery at 10 days after treatment with propolis ointment in combination with ivermectin. (**E**) Untreated control showing alopecia allover the body surface. (**F**) Photomicrograph of *S. scabiei* egg containing larva detected in skin scrapings from rabbit treated with ivermectin (400×).

Based on the clinical signs (Figure 2A,B), healing was noted after 7 days of treatment with 10% propolis ointment (Figure 2C) or through its combination with ivermectin (Figure 2D) with new hair growth and smooth skin observed after the 15^th^ day of treatment. In the group that was treated with ivermectin alone, there was no recovery until the 28^th^ day of treatment. Untreated control rabbits displayed signs of sarcoptic mange during the course of the entire study (Figure 2E). After the 7^th^ day of treatment, skin scrapings from the rabbits of groups II and IV were consisted of dead mites. After 15 days, these skin scrapings were found to be negative for the presence of larvae, eggs, as well as adult parasites. Meanwhile, in group III, dead mites were found during microscopic examinations until the 28^th^ day, whereas the eggs could be detected in skin scrapings that were gathered when the experiment ended (Figure 2F).

### Biochemical analysis

**Figure 3:**
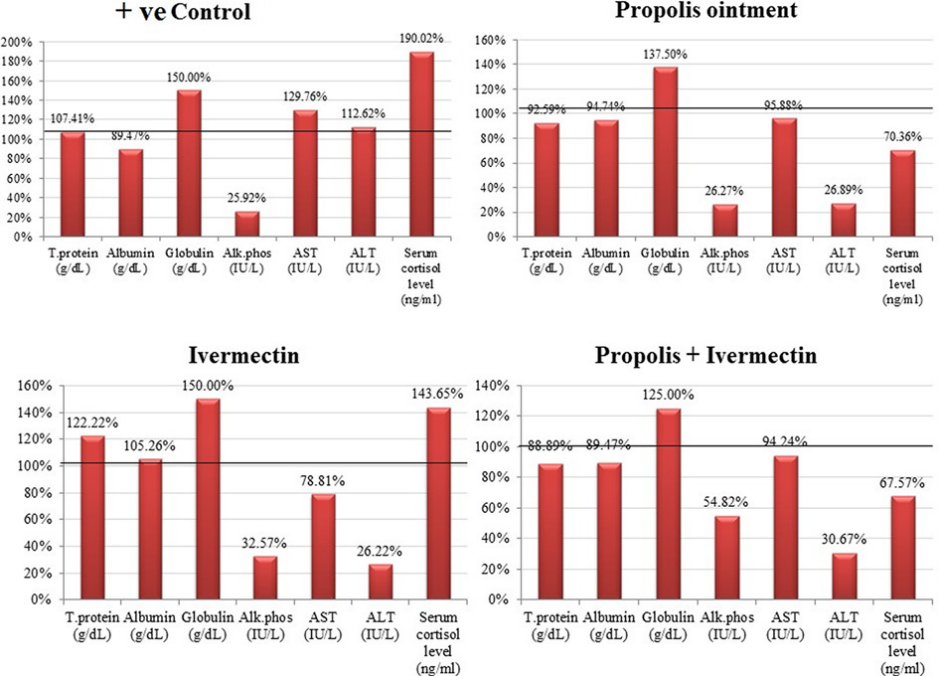
Percentage changes in the total protein, albumin, globulin, ALP, AST, ALT, and serum cortisol in the positive control rabbits (infested but not treated) and infested rabbits treated with 10% propolis ointment, ivermectin, and propolis ointment in combination with ivermectin.

**Figure 4:**
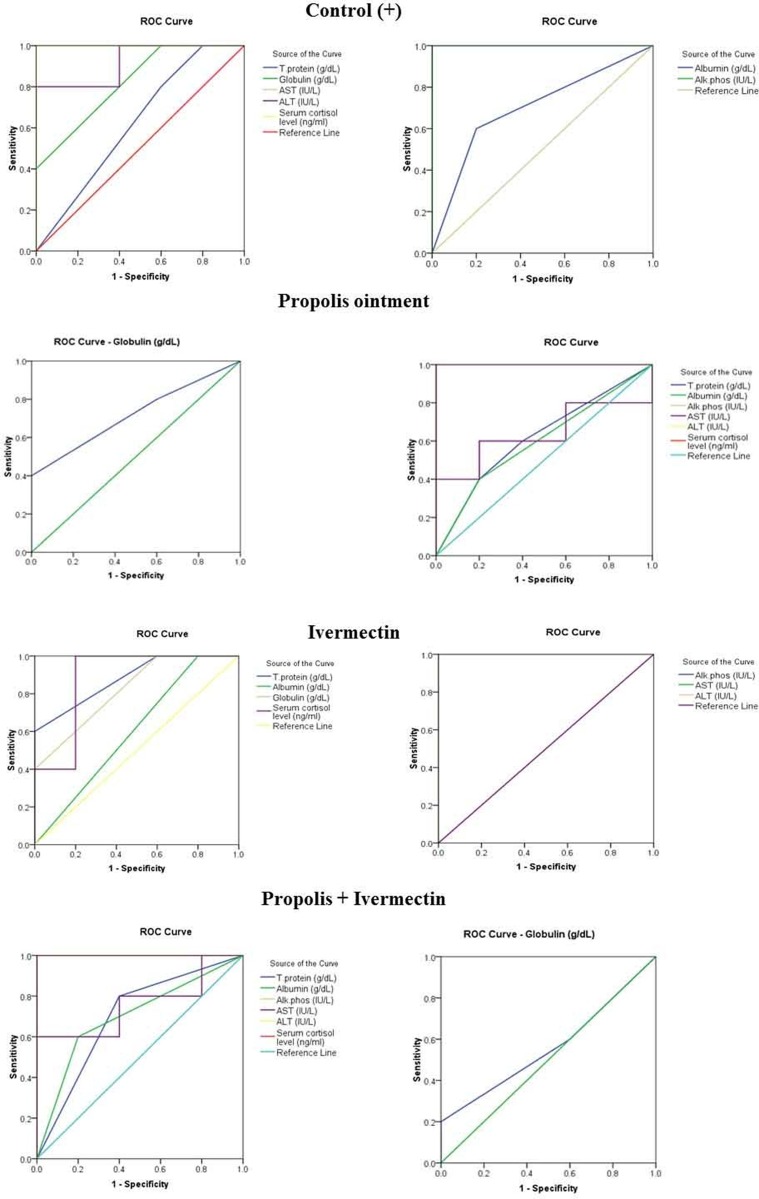
The receiver operating characteristic curve of all parameters in positive control, 10% propolis-treated, ivermectin-treated, and propolis/ivermectin-treated groups

From the group that was treated with ivermectin alone, total protein levels were found to be considerably high. Notably, no major differences were observed in the levels of globulin and albumin in treated groups. ALT, ALP, AST, as well as cortisol were observed to be the most significantly impacted parameters. ALP was significantly reduced in the treated groups. ALT and AST levels increased significantly in the untreated infested control group. AST was reduced significantly in the group that was treated with ivermectin, and ALT decreased significantly in the treated groups. Similarly, cortisol levels increased considerably in the untreated infested control group and the group treated with ivermectin, and it decreased significantly among groups that received the treatments involving 10% propolis ointment (groups II and IV). The change in percentages of the measured parameters within the treated groups and the comparison with control is illustrated in Figure 3. According to the ROC analysis, satisfactory values were determined for sensitivity, specificity and AUC (Table 1 and Figure 4). Our findings revealed that propolis did not produce any aggressive impact on liver factors.

**Table 1:** Receiver operating characteristic curve of all parameters in the positive control, 10% propolis ointment-treated, ivermectin-treated, and propolis ointment/ivermectin combination-treated groups

Parameters	Group	AUC	Cut-off value	Sensitivity	Specificity
**Total protein (g/dl)**	(+ve) Control	0.620	5.500	80.0%	40.0%
	10% propolis ointment	0.620	5.500	60.0%	60.0%
	ivermectin	0.880	6.500	60.0%	100.0%
	propolis + ivermectin	0.700	5.500	80.0%	60.0%
**Albumin (g/dl)**	(+ve) Control	0.700	3.500	60.0%	80.0%
	10% Propolis ointment	0.600	3.500	40.0%	80.0%
	ivermectin	0.600	3.500	100.0%	20.0%
	propolis + ivermectin	0.700	3.500	60.0%	80.0%
**Globulin (g/dl)**	(+ve) Control	0.820	2.500	40.0%	100.0%
	10% propolis ointment	0.720	2.500	40.0%	100.0%
	ivermectin	0.820	2.500	40.0%	100.0%
	propolis + ivermectin	0.560	3.000	20.0%	100.0%
**ALP (IU/l)**	(+ve) Control	1.000	57.500	100.0%	100.0%
	10% propolis ointment	1.000	56.000	100.0%	100.0%
	ivermectin	1.000	63.500	100.0%	100.0%
	propolis + ivermectin	1.000	69.500	100.0%	100.0%
**AST (IU/l)**	(+ve) Control	1.000	55.620	100.0%	100.0%
	10% propolis ointment	0.640	46.900	60.0%	80.0%
	ivermectin	1.000	45.295	100.0%	100.0%
	propolis + ivermectin	0.760	45.950	60.0%	100.0%
**ALT (IU/l)**	(+ve) control	0.920	48.405	80.0%	100.0%
	10% Propolis ointment	1.000	28.460	100.0%	100.0%
	ivermectin	1.000	27.870	100.0%	100.0%
	propolis + ivermectin	1.000	29.510	100.0%	100.0%
**Serum cortisol (ng/ml)**	(+ve) Control	1.000	3.930	100.0%	100.0%
	10% propolis ointment	1.000	2.195	100.0%	100.0%
	ivermectin	0.880	2.840	100.0%	80.0%
	propolis + ivermectin	1.000	2.190	100.0%	100.0%

AUC (area under the curve)

AUC = 0.5–0.65 (useless biomarker)

AUC = 0.7–0.85 (good biomarker)

AUC = 0.86–1 (with satisfactory sensitivity and specificity: excellent biomarker)

## Discussion

Scabies is a common parasitic infestation that is difficult to eradicate [29]. Drugs that are presently used for scabies have several limitations, including resistance development [7], toxicity, long-term damage [8], and practical difficulties in management. All of these challenges have reinforced the need for more effective and safer drugs. Ivermectin is used extensively for scabies. Since the introduction of ivermectin for the treatment of scabies, reports of adverse events have been rare although this drug can cause cardiac dysfunction and hepatitis [29]. Propolis is a nontoxic natural product; however, some cases of allergy and contact dermatitis to this compound have been described, mainly among beekeepers. An important factor in impaired wound healing is biofilm formation. Propolis, as an anti-microbial agent, can reduce biofilm generation and result in accelerated healing. Most of the *in vivo* studies on various wound models suggest the beneficial role of propolis on experimental wound healing and this has also been proven in clinical trial studies. Nevertheless, there is a lack of information concerning dose, side effects and clinical effectiveness of propolis for wounds. [30]. In the present study, it was found that the topical application of 10% propolis ointment effectively killed *S. scabiei* mites. In fact, the rabbits exhibited complete recovery after 15 days of treatment, while rabbits that were treated with ivermectin were observed to recover by the end of this experiment. The findings of this treatment’s success had been previously validated [15,31,32] by researchers using oils. According to the results, the total protein level of the group that was treated with ivermectin was significantly high. This result contradicted a previous study [17] wherein the total protein levels were found to decrease significantly in groups that were treated with ivermectin. The treatment of rabbits with ivermectin in our study revealed a nonsignificant surge in serum albumin. Interestingly, the results were inconsistent with the findings of previous studies among rabbits [18] and rats [19] in which the administration of ivermectin was found to decrease serum albumin significantly. Our results did not reveal any major differences in globulin levels [33], although the findings contradict the results of a previous study [13] in which the rabbits’ serum globulin levels in ivermectin and positive control groups were found to be considerably low, while the albumin and total protein levels were not affected significantly [19]. ALP levels dropped significantly (groups I, II, III, and IV). The underlying reason for reduced ALP levels was partially attributed to the mite infected rabbits’ weight loss, which could be due to the ‘energy demands’ through the production of scratching and parakeratotic scale, along with lower food consumption [34]. ALT and AST levels increased significantly in the infected control group. Moreover, AST decreased considerably in the group treated with ivermectin that was indicative of the negative effects of the hepatic factor. Additionally, ALT decreased considerably in the groups treated with propolis, ivermectin, and a combination of the two compared with the healthy control group. It must be noted that this result was contrary to previous studies carried out in rats [28], rabbits [2,35] and rams, as well as bucks [36], wherein the groups treated with ivermectin experienced impaired liver functions and inflammation of liver cells. Serum ALT and AST levels in cattle and swine rose significantly after 28 days of treatment with ivermectin [36]. Since cortisol is a significant hormone that is released as a response to stress [37], it is often measured to evaluate overall welfare and stress [38,39]. Cortisol levels in rabbits went up significantly in ivermectin-treated and untreated infested control group. These levels in groups treated with propolis/ivermectin and propolis alone are decreased significantly in comparison with the untreated infested control group. The ROC analysis for ALP, AST, ALT, and cortisol showed satisfactory specificity and sensitivity along with a heightened AUC of 1. As a result, we postulate that such parameters were feasible biomarkers in the context of following the diseased rabbits’ response to the therapeutic impact of propolis.

This investigation established the anti-sarcoptic effects (*in vivo*) of propolis and proposed the evaluation of particular biomarkers for use as predictive tools for improving treatment outcomes. Additional experiments with different application methods and concentrations to assess the efficacy of propolis in healing/treating sarcoptic mange lesions are needed.

## Abbreviations

ALP: alkaline phosphatase
ALT: alanine amino transferase
AST: aspartate amino transferase
AUC: area under the curve
ROC: receiver operating characteristic
